# Clinical Analysis of Comprehensive Pharmacotherapy Combined With Ultrasound‐Guided Precise Lesion Resection Plus Primary Microplasty in the Treatment of Nonpuerperal Mastitis

**DOI:** 10.1155/tbj/4928115

**Published:** 2026-05-28

**Authors:** Bao-Zhong Yao, Kun Peng, Sai-Long Sang, Li Lin, Hong-Cun Chen, Hong-Lin Li, Dai-Wei Shi, Liang Li, Qi-Ru Xiong

**Affiliations:** ^1^ Department of General Surgery, The Second Affiliated Hospital of Anhui Medical University, Hefei, 230060, Anhui, China, ahmu.edu.cn; ^2^ Department of Thyroid and Breast Surgery, The Second People’s Hospital of Hefei (Anhui Medical University Affiliated Hefei Hospital), Hefei, 230011, Anhui, China

**Keywords:** breast microplasty, comprehensive pharmacotherapy, cosmetic outcome, nonpuerperal mastitis, precise lesion resection, recurrence rate

## Abstract

**Objective:**

To explore the clinical efficacy, cosmetic outcome, and safety of comprehensive pharmacotherapy (traditional Chinese medicine + hormone + antibiotic) combined with ultrasound‐guided precise lesion resection plus primary microplasty in the treatment of refractory nonpuerperal mastitis (NPM).

**Methods:**

The clinical and pathological data of refractory NPM patients who underwent surgical treatment at our hospital from February 2021 to December 2024 were retrospectively analyzed. The sample size was calculated using a superiority test, and a total of 97 patients were finally included. They were assigned to two groups using a random number table for retrospective stratification assignment (to balance baseline clinical characteristics and reduce selection bias): The control group (45 cases) underwent extended lesion resection combined with fascial flap plasty and nipple–areola correction and the observation group (52 cases) underwent ultrasound‐guided precise lesion resection plus primary microplasty. The recurrence rate, breast cosmetic score (Harris score), postoperative psychological status (24‐item Hamilton Depression Rating Scale [HAMD‐24]), hospital stay, and incidence of complications were compared between the two groups.

**Results:**

All surgeries were successfully completed in both groups without serious complications. All patients were followed up for more than 12 months (median follow‐up period: 16.8 ± 2.9 months). There were no statistically significant differences in the incidence of postoperative complications [7.7% (4/52) vs. 6.7% (3/45), *p* = 1], drainage time [(3.5 ± 0.3) d vs. (3.6 ± 0.4) d, *t* = −1.398, *p* = 0.166], and hospital stay [(10.7 ± 0.6) d vs. (10.6 ± 0.5) d, *t* = 0.894, *p* = 0.373] between the two groups. The recurrence rate of the observation group was lower than that of the control group [0% (0/52) vs. 11.1% (5/45), *X* ^2^ = 9.836, *p* = 0.002], and the cosmetic effect and patient satisfaction of the observation group were superior to those of the control group. The HAMD‐24 score of the observation group [(7.8 ± 2.9) points] was lower than that of the control group [(12.5 ± 3.8) points], with a statistically significant difference (*t* = 11.562, *p* = 0.001).

**Conclusion:**

For refractory NPM, the combination of comprehensive pharmacotherapy, ultrasound‐guided precise lesion resection, and primary microplasty achieves remarkable therapeutic effects, characterized by reduced complication rates and low short‐to‐long‐term recurrence. This integrated traditional Chinese and Western medicine strategy is not only safe and effective but also provides excellent cosmetic benefits for patients.

## 1. Introduction

Nonpuerperal mastitis (NPM) is a spectrum of benign chronic inflammatory disorders predominantly affecting nonlactating women of age 20–58 years, primarily encompassing two pathological subtypes: granulomatous lobular mastitis (GLM) and plasma cell mastitis (PCM) [1, 2]. Its typical clinical manifestations include breast masses, pain, abscess formation, and sinus tract/fistula development, which are characterized by a protracted disease course and high recurrence rate. In recent years, the global incidence of NPM has exhibited an upward trend, accounting for 2%–5% of all breast lesions in China, and it has thus become a significant challenge in the diagnosis and management of breast diseases [3].

Currently, multiple therapeutic modalities are available for NPM, yet no unified clinical consensus has been established [4]. Commonly utilized medications include antibiotics, glucocorticoids, methotrexate, and traditional Chinese medicine (TCM) [5–7]. Among these, antibiotics can control acute inflammation but are rarely curative [8]; glucocorticoid therapy yields certain therapeutic effects, though long‐term high‐dose administration may lead to severe complications such as delayed wound healing [9]; TCM treatment is associated with limitations including slow onset of action and prolonged treatment duration, which easily impose substantial economic and psychological burdens on patients [10].

Surgical intervention enables direct resection of inflammatory lesions; however, traditional extended lesion resection or mastectomy often compromises breast aesthetics, with postoperative recurrence rates ranging from 1% to 50% [2, 11]. With the advancement of minimally invasive technologies, the management of NPM has gradually progressed toward precision and cosmetic optimization. Oncoplastic surgery (OS) integrates the advantages of oncological resection and plastic reconstruction. Originating from the demand for improved postoperative breast cosmesis among breast cancer patients, OS has been widely applied in the treatment of various breast diseases with favorable outcomes [12–14].

In light of the limitations of existing treatment regimens, our medical team integrated the strengths of TCM and Western medicine to propose a novel therapeutic strategy for NPM: “comprehensive pharmacotherapy (TCM + glucocorticoids + antibiotics) combined with innovative plastic surgery (ultrasound‐guided precise lesion resection + primary oncoplastic reconstruction).” Utilizing a retrospective cohort study design, this study systematically evaluates the clinical efficacy, cosmetic outcomes, and safety of this combined regimen in the treatment of refractory NPM, aiming to provide evidence‐based references for the standardized management of refractory NPM. The present study was reported in strict adherence to the STROCSS 2019 Guidelines [15] and the TITAN Guideline Checklist 2025 [16]. Additionally, we followed the STROBE statement for reporting observational studies [17].

## 2. Materials and Methods

### 2.1. Study Population

Based on the superiority test for two independent sample rates (*α* = 0.05, *β* = 0.2, preset follow‐up loss rate of 20%, and 1:1 sample size ratio between groups), 34 patients were required per group. Our center admits approximately 50 NPM patients undergoing surgery annually, among whom refractory NPM (characterized by extensive lesions involving two or more quadrants, or complicated with abscess, sinus tract/fistula, skin ulceration, or failure of ≥ 4 weeks of conservative medical treatment with antibiotics/steroids) accounts for about 80%. A total of 97 refractory NPM patients were enrolled between February 2021 and December 2024 in accordance with the inclusion and exclusion criteria, ensuring a sufficient sample size. All patients signed the informed consent forms for both the study and surgery and were assigned to the observation group (*n* = 52) and the control group (*n* = 45) using a random number table for retrospective stratification assignment (to balance baseline clinical characteristics such as lesion range and complication types, and reduce selection bias in retrospective studies).

According to clinical and ultrasound findings, the observation group was classified into four subtypes [18]: Type I (single mass), Type II (single quadrant with or without 1 skin lesion), Type III (multiple quadrants with or without 1 skin lesion), and Type IV (multiple quadrants with multiple skin lesions). Patients in the observation group underwent ultrasound‐guided precise lesion resection combined with primary oncoplastic reconstruction, whereas those in the control group received extended lesion resection combined with fascial flap plasty and nipple–areola correction. No statistically significant differences were observed in preoperative general data between the two groups (Table 1).

**TABLE 1:** Comparison of general characteristics between patients undergoing ultrasound‐guided precise lesion resection + primary oncoplastic reconstruction (observation group) and those undergoing extended lesion resection + fascial flap plasty + nipple–areola correction (control group).

Characteristics	Control group (*n* = 45)	Observation group (*n* = 52)	Statistic	*p* value
*Clinical presentation (n)*				
Breast mass	45	52	—	—
Breast pain	40	48	—	0.729^∗^
Axillary lymphadenopathy	20	28	*χ* ^2^ = 0.853	0.356
Nipple discharge	10	12	*χ* ^2^ = 0.010	0.921
Inflammatory skin changes	30	43	*χ* ^2^ = 3.327	0.068

*Affected side (n)*				
Right	18	24	*χ* ^2^ = 0.372	0.542
Left	22	25	*χ* ^2^ = 0.006	0.940
Bilateral	5	3	—	0.466^∗^

*Previous pregnancies (n)*				
0	3	5	—	0.721^∗^
1	13	11	*χ* ^2^ = 0.775	0.379
2	25	29	*χ* ^2^ = 0.000	0.983
≥ 3	4	7	—	0.537^∗^

*Previous deliveries (n)*				
0	3	6	—	0.498^∗^
1	35	36	*χ* ^2^ = 0.898	0.343
≥ 2	7	10	*χ* ^2^ = 0.225	0.635
History of breastfeeding difficulties (*n*)	38	44	*χ* ^2^ = 0.001	0.981
History of acute mastitis (*n*)	13	20	*χ* ^2^ = 0.985	0.321
Age (years), mean ± SD	33.7 ± 5.8	33.3 ± 7.0	*t* = 0.304	0.762
Arthralgia (*n*)	0	0	—	1^∗^
Erythema nodosum (*n*)	4	8	—	0.373^∗^
History of oral contraceptives (*n*)	15	19	*χ* ^2^ = 0.109	0.741
History of fibroadenoma (*n*)	5	10	*χ* ^2^ = 1.217	0.270
History of breast surgery (*n*)	2	2	—	1^∗^
Pre‐existing nipple inversion (*n*)	21	28	*χ* ^2^ = 1.268	0.260
History of breast trauma (*n*)	13	19	*χ* ^2^ = 0.639	0.424
Smoking (active/passive) (*n*)	7	15	*χ* ^2^ = 2.430	0.119

*Note:* The *P* value obtained by Fisher’s exact test.

^∗^— indicates no data.

All surgeries were performed by two senior endocrine surgeons with more than 10 years of clinical experience (certified by the Chinese Society of Thyroid and Breast Surgery) to ensure operational consistency. The study was approved by the Institutional Review Board and conducted in strict compliance with the Declaration of Helsinki (2013 revision). Written informed consent was obtained from all patients or their legally authorized representatives.

### 2.2. Study Design and Protocol

This retrospective cohort study was designed and reported in accordance with the STROCSS 2019 Guideline Checklist for cohort studies.

#### 2.2.1. Inclusion Criteria

Inclusion criteria were defined as follows: ① nonlactating women of age 18–60 years without a history of prior relevant surgical treatment; ② histopathologically confirmed NPM (including GLM and PCM); ③ refractory NPM: lesions involving one or more breast quadrants, or complicated with complex lesions such as abscess, sinus tract/fistula formation, or skin ulceration, or failure of ≥ 4 weeks of conservative medical treatment (antibiotics/steroids); and ④ voluntary participation in the study and signing of the informed consent form.

#### 2.2.2. Exclusion Criteria

Exclusion criteria were defined as follows: ① follow‐up duration less than 12 months or incomplete follow‐up data; ② severe heart failure, uncontrolled chronic respiratory diseases, or coagulation disorders, making the patient unable to tolerate surgery or refusing surgery; ③ complicated with uncontrolled infections of any type or surgical contraindications; ④ complicated with other breast diseases, such as breast cancer and breast fibroadenoma; ⑤ history of long‐term oral risperidone or other antipsychotic medications; and ⑥ male patients.

#### 2.2.3. Preoperative Preparation

Patients in the observation group received preoperative treatment with TCM (Xiaojin capsules) + antibacterial agents + dexamethasone for 5–7 days, while those in the control group received antibacterial agents + dexamethasone for 5–7 days. Both groups continued oral dexamethasone for 1 month postoperatively with a tapering dosage: 1 tablet/time, 4 times/day in the first week; 1 tablet/time, 3 times/day in the second week; 1 tablet/time, 2 times/day in the third week; and 1 tablet/time, 1 time/day in the fourth week. Additionally, the observation group took Xiaojin capsules orally for 3 months (4 capsules/time, 3 times/day).

Patients with elevated preoperative prolactin levels underwent re‐examination every 3 months postoperatively, and low‐dose bromocriptine was administered if necessary. Postoperative complications such as delayed wound healing, bleeding, hematoma, infection, and fat liquefaction were recorded throughout the follow‐up period.

### 2.3. Surgical Procedure

#### 2.3.1. Observation Group

Ultrasound‐guided precise lesion resection combined with primary oncoplastic reconstruction was performed. Patients were placed under general endotracheal anesthesia and in a supine position with the affected upper limb abducted. Preoperative ultrasound was used to localize the lesion range and design the incision (arc‐shaped incision along the areola, extending approximately 4 cm to both sides) into the following types: Type I: Direct precise lesion resection was performed and Types II, III, and IV: The incision was symmetrically extended to both sides along the areola. The gland was incised to the retromammary space using an electric scalpel, and the entire gland was flipped upward. Intraoperative ultrasound exploration was performed to identify residual lesions. Necrotic tissue was resected with surgical scissors, and tiny abscesses were cleared with a curette while preserving normal glandular tissue as much as possible. In the observation group, intraoperative ultrasound exploration was performed to identify residual and occult microlesions, which can effectively reduce the risk of missed resection of multicentric lesions.

After complete lesion clearance, the surgical field was sequentially irrigated with 3% hydrogen peroxide solution, 1% povidone–iodine, and normal saline, with special attention to irrigating the lesion base, incision margins, and lactiferous ducts. The wound surface was soaked in 1% povidone–iodine for 10 min and then irrigated with normal saline until the effluent was clear. Adjacent flap transfer technology was used to eliminate the residual glandular cavity, or primary repair procedures such as inverted T‐shaped breast reduction plasty and areola lifting were performed. When handling the nipple–areola complex, an appropriate thickness of glandular tissue was preserved, necrotic tissue was thoroughly curetted, and double‐layer purse‐string suturing was performed at the nipple base to prevent inversion.

#### 2.3.2. Control Group

Patients in the control group underwent extended lesion resection combined with fascial flap plasty and nipple–areola correction. The anesthesia method and patient position were the same as those in the observation group. An incision was made along the Langer’s lines of the skin, with the length determined by the lesion range. An extended resection was performed within an area more than 2 cm away from the lesion tissue to ensure negative pathological margins for granulomatous lesions in the postoperative specimen. Surgical field irrigation was identical to that in the observation group, followed by fascial flap plasty and nipple–areola correction.

#### 2.3.3. Postoperative Drainage

**FIGURE 1:**
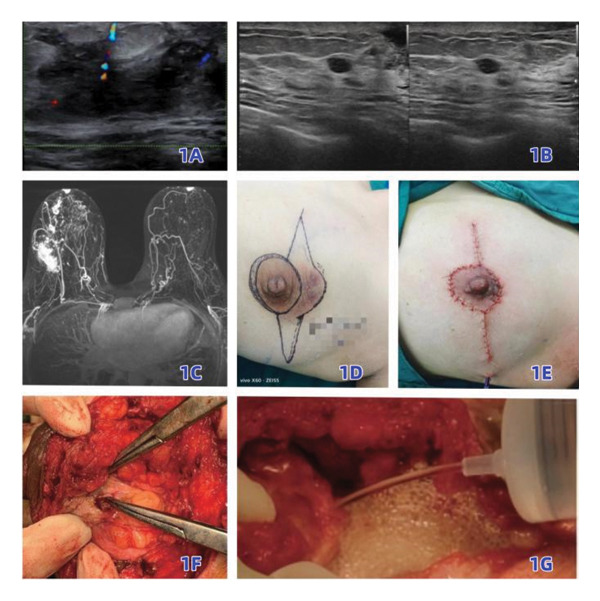
Images related to ultrasound‐guided precise lesion resection + primary oncoplastic reconstruction: preoperative ultrasound examination for lesion localization (1A); intraoperative ultrasound repositioning of tiny lesions (1B); preoperative breast MRI (1C); preoperative breast lesion (1D); primary oncoplastic reconstruction + postoperative drainage (1E); curette removal of tiny lesions and precise resection of necrotic tissue (1F); intraoperative irrigation (1G).

**FIGURE 2:**
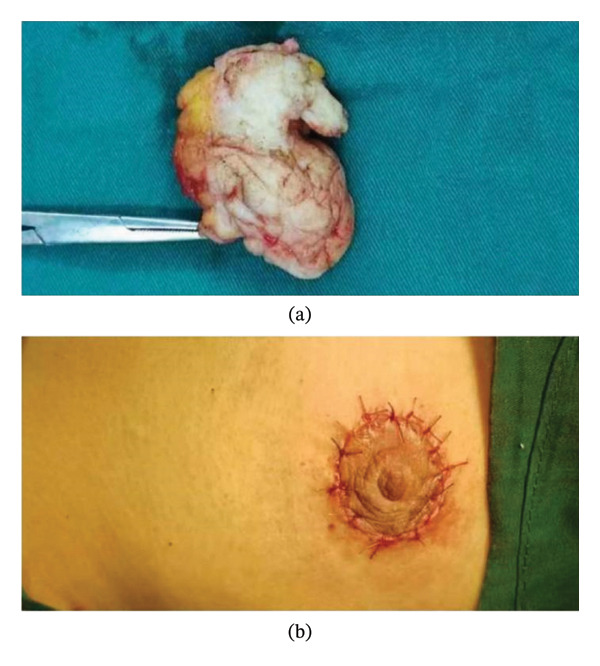
Images related to extended lesion resection combined with fascial flap plasty and nipple–areola correction: extended resection of the lesion (a); Nipple–areola correction (b).

A drainage tube was placed in the surgical area of all patients and exteriorized from the midaxillary line through a subcutaneous tunnel. Dressing changes were performed every other day postoperatively, and the nature and volume of drainage fluid were closely observed. The drainage tube was removed when the 24‐h drainage volume was less than 10 mL (Figures 1 and 2).

### 2.4. Observation Indicators

#### 2.4.1. Follow‐Up and Recurrence

Follow‐up was conducted through outpatient re‐examination, telephone, and WeChat. Postoperative visits were scheduled on Day 14, Month 1, Month 3, and then every 3 months for 1 year. Physical examination, breast ultrasound, and questionnaires were performed every 3 months.

*Cure criteria*: (all maintained for > 1 year): ① disappearance of breast pain/swelling; ② no skin redness, ulceration, erythema nodosum, or joint pain on palpation; and ③ no abnormal echo on ultrasound

*Recurrence criteria*: Reappearance of inflammatory lesions (mass, abscess, or fistula) in the ipsilateral breast within 1 year postsurgery, with or without systemic symptoms

#### 2.4.2. Postoperative Complications

Drainage time, hospital stay, and complications (fat liquefaction, delayed healing, hematoma, and nipple ischemia/necrosis) were recorded and compared.

#### 2.4.3. Cosmetic Outcome and Satisfaction

Breast appearance was evaluated using the Harris criteria [19] (Table 2). They included total scores as follows: 18–20 = excellent, 14–17 = good, 10–13 = fair, and < 10 = poor. Patient satisfaction (very satisfied, satisfied, or dissatisfied) was assessed through a questionnaire at 1 year postsurgery.

**TABLE 2:** Harris criteria for the evaluation of breast appearance and cosmetic outcomes.

Item	1 point	2 points	3 points	4 points
Nipple lateral displacement	> 3.0 cm	1.5–3.0 cm	0–1.5 cm	No displacement
Degree of local retraction (affected breast)	Marked retraction	Moderate retraction	Mild retraction	No retraction
Surgical scar	Hypertrophic/obvious	Clearly different from normal skin	Minimally different from normal skin	Concealed
Bilateral breast symmetry	Marked asymmetry	Moderate symmetry	Good symmetry	Excellent symmetry
Skin texture (affected breast)	Poor elasticity	Reduced elasticity	Slight change	No change

### 2.5. Statistical Analysis

Statistical analysis was performed using the SPSS 28.0 software. Measurement data conforming to a normal distribution were expressed as mean ± standard deviation (*x̄* ± *s*), and comparisons between groups were conducted using the independent samples *t*‐test. Count data were expressed as cases (percentages). The chi‐square test or Fisher’s exact test was used for comparisons of unordered categorical data between groups, and the Wilcoxon rank‐sum test was used for comparisons of ordered categorical data between groups. A *p* value < 0.05 was considered statistically significant.

## 3. Results

### 3.1. Safety Evaluation

All surgeries were successfully completed in both groups without serious complications. The vast majority of complications in both groups were Grade I or Grade II, which could be relieved without intervention or with only minimal medication (postoperative complications based on the Clavien–Dindo classification system). The incidence of complications in the observation group was 7.7% (4/52), including 3 cases of fat liquefaction and 1 case of nipple ischemia/necrosis; the incidence of complications in the control group was 6.7% (3/45), all of which were fat liquefaction. There were no statistically significant differences in postoperative drainage time, hospital stay, and overall complication rate between the two groups (all *p* > 0.05) (Table 3).

**TABLE 3:** Comparison of surgical outcomes between the observation group and the control group.

Outcome measures	Observation group (*n* = 52)	Control group (*n* = 45)	Statistic	*p* value
Drainage time (days), mean ± SD	3.5 ± 0.3	3.6 ± 0.4	*t* = −1.398	0.166
Hospital stay (days), mean ± SD	10.7 ± 0.6	10.6 ± 0.5	*t* = 0.894	0.373
Postoperative complications [*n*/*N* (%)] (Clavien–Dindo grade)				
Overall complications	4/52	3/45		1.00^∗^
Grade I				
Fat liquefaction	3/52	3/45		
Grade II				
Nipple ischemia/necrosis	1/52	0/45		
Grades III–V	0/52	0/45		
Recurrence (*n*)	0	5		0.02
Cosmetic outcome (*n*)			*Z* = −3.012	0.003
Excellent	17	10		
Good	28	18		
Fair + poor	7	17		
Patient satisfaction (*n*)			*Z* = −5.824	< 0.001
Very satisfied	18	13		
Satisfied	29	12		
Dissatisfied	5	20		

*Note:* The *P* value obtained by Fisher’s exact test.

^∗^— indicates no data.

### 3.2. Recurrence

All patients completed follow‐up for more than 12 months, with a median follow‐up time of (16.8 ± 2.9) months. There was no recurrence in the observation group (0/52), while 5 cases (11.1%) reported recurrence in the control group, with a recurrence time of 45–72 days (average 53.6 ± 4.2 days). The difference in the recurrence rate between the two groups was statistically significant [0% (0/52) vs. 11.1% (5/45), *p* = 0.02].

### 3.3. Cosmetic Outcome and Satisfaction

**FIGURE 3:**
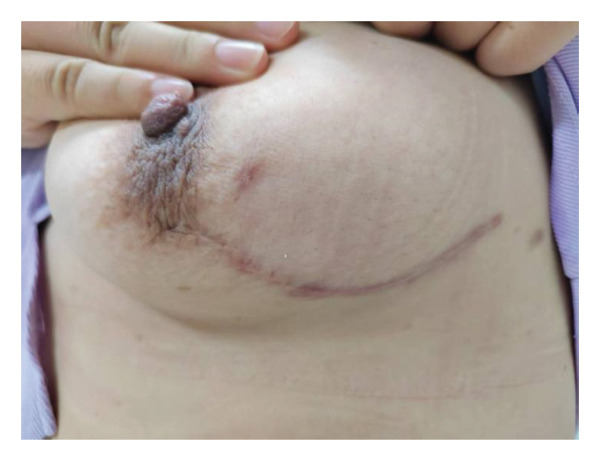
Satisfactory breast appearance 1 year after surgery.

The breast cosmetic effect score of the observation group was better than that of the control group, and the excellent and good rate (excellent + good) was higher [86.5% (45/52) vs. 62.2% (28/45), *Z* = −3.012, *p* = 0.003]. The 1‐year postoperative satisfaction survey showed that the satisfaction of the observation group was better than that of the control group [90.3% (47/52) vs. 55.6% (25/45), *Z* = −5.824, *p* < 0.001] (Table 3, Figure 3).

### 3.4. Comparison of Psychological Status Between the Two Groups

Six months after surgery, the 24‐item Hamilton Depression Rating Scale (HAMD‐24) score of 52 patients in the observation group was (7.8 ± 2.9) points, which was lower than that of 45 patients in the control group (12.5 ± 3.8) points, with a statistically significant difference (*t* = 11.562, *p* < 0.001) (Table 4).

**TABLE 4:** Comparison of HAMD‐24 scores between the two groups at 6 months postoperation (*x̄* ± *s*).

Group	Item	HAMD‐24 score (points)
Observation group (*n* = 52)	52	7.8 ± 2.9
Control group (*n* = 45)	45	12.5 ± 3.8
*t*‐value		11.562
*p* value		< 0.001

## 4. Discussion

NPM mainly includes GLM and PCM. It is a spectrum of chronic, recurrent inflammatory breast disorders predominantly affecting women of childbearing age. In recent years, its incidence has shown an upward trend, making it a significant challenge in the diagnosis and treatment of breast diseases [20]. The clinical manifestations of this disease are complex and diverse, ranging from occult solitary masses to rapidly progressing diffuse inflammation accompanied by redness, swelling, heat, and pain, and even the formation of refractory abscesses or cutaneous sinuses. Its imaging features often overlap with those of breast cancer, which not only increases the difficulty of diagnosis but also causes significant psychological anxiety in patients [21, 22]. The fundamental etiology of NPM remains unclear to date, with interweaving hypotheses such as autoimmune abnormalities, excessive inflammatory responses to occult pathogens (e.g., *Corynebacterium*), hyperprolactinemia, and ductal emptying dysfunction forming a complex pathogenic network [23, 24]. This ambiguity in etiology directly leads to the lack of a globally recognized and unified treatment plan. Achieving an effective balance between thorough lesion clearance, reducing recurrence rate, preserving breast function and aesthetics, and improving patients’ psychological status has become a key issue that needs to be addressed urgently in clinical breast surgery.

We acknowledge that NPM can often be managed successfully with medical therapy alone, including local or systemic glucocorticoids and antibiotics, particularly in the early solid or cavitary phases. As highlighted by recent studies [25, 26], the disease has a natural course progressing from solid lesions to thin‐walled cysts and eventually scar formation, and many patients achieve healing without surgery. However, the patient population in our study was highly selected: All had failed prior medical therapy (≥ 4 weeks of antibiotics and/or steroids) or presented with extensive lesions (≥ 2 quadrants), abscesses, sinus tracts, or skin ulceration—features that are less likely to respond to medication alone and often lead to prolonged suffering and poor cosmetic outcomes if left unoperated. For such refractory cases, we advocate a *comprehensive approach* that combines preoperative pharmacotherapy (to reduce inflammation and lesion burden) with *ultrasound-guided precise lesion resection* (to remove residual nidi while preserving healthy tissue) and *primary oncoplastic reconstruction* (to restore breast contour). This strategy is not a replacement for medical therapy in mild disease but an option for selected patients who would otherwise face mastectomy or chronic disability. Our results demonstrate that with this approach, recurrence can be reduced to 0% (in the observation group) while achieving high cosmetic satisfaction, without increasing complications.

The limitations of traditional treatment models have become increasingly prominent in long‐term clinical practice. For a small number of cases with localized lesions, mild symptoms, and a self‐limiting course, close observation and follow‐up are reasonable strategies. Some studies have reported that patients can achieve spontaneous healing within 2 years of onset without intervention [27, 28]. However, for patients with obvious symptoms or continuously expanding lesions, simple observation and follow‐up mean enduring prolonged suffering and facing the risks of breast deformity and complex fistula formation due to disease progression. Pharmacotherapy is an important treatment for NPM. Systemic glucocorticoids are widely used due to their potent anti‐inflammatory and immunosuppressive effects, with a short‐term clinical response rate of up to 72%. Nevertheless, they present a dual dilemma: First, the recurrence rate after drug withdrawal is high, with approximately 21% of patients experiencing recurrence within 2 years of withdrawal; second, long‐term use is prone to adverse reactions such as metabolic disorders, osteoporosis, and increased infection risk, which seriously affect patient compliance and quality of life [29]. The therapeutic regimen of methotrexate combined with low‐dose corticosteroids has shown promising efficacy. A study involving 62 patients with idiopathic granulomatous mastitis (IGM) demonstrated that the success rate of this regimen was 93.71%, with a mean remission period of 10.14 months [30]. In addition, the triple antituberculosis drug therapy has provided a new option for NPM treatment. A study treating 22 GLM patients with rifampicin, isoniazid, and ethambutol showed that 18 patients achieved complete remission with no recurrence during long‐term follow‐up [31]. TCM has demonstrated unique advantages in NPM treatment, and its concepts of holistic syndrome differentiation and individualized treatment are highly consistent with the complex pathogenesis of NPM. Xiaojin capsule, a classic Chinese patent medicine, possesses the effects of promoting blood circulation to unblock meridians and resolving masses to reduce swelling. Modern pharmacological studies have confirmed that it also has definite anti‐inflammatory and immunomodulatory effects, which can effectively reduce lesion volume and control inflammatory responses [10]. The comprehensive pharmacotherapeutic regimen of Xiaojin capsules combined with hormones and antibiotics adopted in this study is based on the therapeutic concept of the synergistic effect between TCM and Western medicine. Preoperatively, hormones and antibiotics are used to rapidly control acute inflammation, while Xiaojin capsule gradually reduces lesions by improving microcirculation and regulating immune function. The synergy of the three reduces surgical difficulty, minimizes intraoperative trauma, and creates favorable conditions for precise surgery.

Surgical treatment is an important radical method for NPM, with the core goal of thoroughly removing lesions to reduce recurrence risk. However, traditional surgical procedures have obvious limitations. As a classic surgical method, the recurrence rate of extended lesion resection is still 20%–30%, mainly due to unclear boundaries of diseased tissue and missed resection of potential lesions. Excessive resection of glands to pursue “negative surgical margins” often comes at the cost of sacrificing the natural contour of the breast, leading to irreversible cosmetic defects, such as breast depression and nipple displacement, which seriously affect patients’ quality of life [13]. Although radical mastectomy can reduce the recurrence rate to a certain extent, it results in breast loss, which is poorly accepted psychologically, especially by young NPM patients [11]. The control group (CG, *n* = 45) in this study underwent extended lesion resection combined with fascial flap plasty and nipple–areola correction, and the postoperative recurrence rate was still 11.1% (5/45), which is consistent with the recurrence rates reported in traditional surgical cohorts.

In recent years, innovations and optimizations in surgical techniques have brought breakthrough progress in NPM treatment. The clinical application of ultrasound localization technology has achieved precise lesion positioning and real‐time intraoperative monitoring, providing technical support for thorough lesion clearance [32, 33]. Rotational glandular anatomy, which involves radial incision of the gland centered on the nipple and rotational exposure of the lesion area, can thoroughly remove scattered microlesions and intraductal secretions. Combined with irrigation with 3% hydrogen peroxide solution, it can release reactive oxygen species and improve the local immune microenvironment. A single‐center retrospective study showed that the median follow‐up recurrence rate of this surgical procedure was only 3.1% at 56 months, and 85.27% of patients were satisfied with the breast appearance [34].

The application of Stage I oncoplastic breast reconstruction is another important innovation of this treatment plan, reflecting the requirements of modern medical humanistic care and the “bio‐psycho‐social” medical model [12]. For NPM patients with different classifications, the formulation of individualized plastic repair strategies is particularly crucial. According to the lesion range, NPM can be divided into four types: Type I (single mass) and Type II (single quadrant lesion) can achieve good breast contour results through complete lesion resection combined with adjacent flap transfer; for refractory patients with Type III (multiquadrant lesions) and Type IV (multiquadrant lesions with multiple skin lesions), conventional surgery is prone to missed lesions and severe breast contour damage, requiring plastic techniques such as volume replacement or volume substitution on the basis of thorough lesion clearance [13]. A randomized controlled study showed that the recurrence rate of the treatment group receiving “lesion clearance combined with total breast exploration and irrigation plus Stage I oncoplastic breast reconstruction” was significantly lower than that of the control group, with higher patient satisfaction [18].

Against this background, the treatment paradigm of NPM urgently needs to be innovated, shifting from a single, passive “confrontational treatment” to an active, integrated “whole‐course management strategy.” Meanwhile, the combination of OS and minimally invasive concepts has also become a treatment trend. The integrated plan of “comprehensive pharmacotherapy (TCM + glucocorticoids + antibiotics) combined with ultrasound‐guided precise lesion resection and Stage I oncoplastic breast reconstruction” proposed and verified in this study is a concentrated manifestation of this concept. The observation group (OG, *n* = 52) adopted ultrasound‐guided precise lesion resection technology. Preoperatively, high‐frequency ultrasound was used to clarify the lesion range and distribution of microlesions, and real‐time intraoperative guidance was provided for lesion resection. This not only ensured thorough removal of diseased tissue but also maximally preserved normal breast tissue, effectively reducing the recurrence risk. With a postoperative follow‐up of more than 12 months (median follow‐up period: 16.8 ± 2.9 months), the recurrence rate of the OG was 0% (0/52), which was significantly lower than that of the CG (11.1%, 5/45), with a statistically significant difference (*p* = 0.02). This fully confirms the important role of precise surgery in reducing the recurrence rate. At the same time, after ultrasound‐guided precise lesion resection, the OG adopted Stage I oncoplastic reconstruction techniques, such as adjacent flap transfer, areola plasty, and double‐layer purse‐string suture of the nipple base, according to the breast defect, which effectively repaired tissue defects, maintained breast shape symmetry, and avoided breast deformities caused by traditional surgery. The study results showed that the breast appearance cosmetic score (Harris score) of the OG was significantly higher than that of the CG, and patient satisfaction was significantly improved, fully reflecting the important value of Stage I oncoplastic reconstruction in improving breast appearance.

The improvement of patients’ postoperative psychological status is one of the important indicators to evaluate the therapeutic effect. As a chronic and refractory disease, NPM is characterized by a long course and high recurrence rate. Coupled with factors such as changes in breast appearance, it often leads to negative emotions such as anxiety and depression in patients, seriously affecting their quality of life [35]. This study used HAMD‐24 to evaluate patients’ postoperative psychological status. The results showed that the HAMD‐24 score of the OG [(7.8 ± 2.9) points] was significantly lower than that of the CG [(12.5 ± 3.8) points], with a statistically significant difference (*t* = 11.562, *p* = 0.001). This indicates that the comprehensive pharmacotherapy combined with ultrasound‐guided precise lesion resection + Stage I oncoplastic breast reconstruction plan can not only effectively treat the disease but also significantly alleviate patients’ negative emotions and improve their mental health by improving breast appearance and reducing recurrence risk, which is highly consistent with the modern medical concept focusing on both physical and psychological rehabilitation.

In terms of treatment safety, all patients in both groups successfully completed the surgery without serious complications. The incidence of postoperative complications in the OG was 7.7% (4/52), and that in the CG was 6.7% (3/45), with no statistically significant difference between the two groups (*p* = 1). The drainage time of the OG was (3.5 ± 0.3) days, and the hospital stay was (10.7 ± 0.6) days; the drainage time of the CG was (3.6 ± 0.4) days, and the hospital stay was (10.6 ± 0.5) days. There were no statistically significant differences between the two groups (*t* = −1.398, *p* = 0.166; *t* = 0.894, *p* = 0.373). These results indicate that although the OG adopted more sophisticated ultrasound localization and oncoplastic techniques, it did not increase surgical trauma and complication risks, nor prolong the hospital stay, fully confirming the safety and feasibility of this treatment plan. The possible reasons are closely related to the effective control of inflammation and reduction of lesions by preoperative comprehensive pharmacotherapy, the reduction of normal tissue damage by precise intraoperative operation, and standardized postoperative care.

The risk factors for NPM recurrence are complex. In addition to incomplete surgical resection, prolactin level, obesity, follicle‐stimulating hormone to luteinizing hormone (FSH/LH) ratio, and tissue IgG4 expression level are all closely related to recurrence [36, 37]. A retrospective analysis of 130 GLM patients found that prolactin level after treatment was higher than that before treatment, which was an independent risk factor for disease recurrence [37]. Another study showed that the high tissue IgG4 expression group (≥ 11 positive cells/high‐power field) had a high initial response rate but a high short‐term recurrence rate after drug withdrawal. It is recommended to add immunosuppressants for maintenance treatment after steroid therapy [37]. In the comprehensive pharmacotherapeutic regimen adopted in this study, Xiaojin capsules have an immunomodulatory effect, and hormones and antibiotics can effectively control inflammatory responses. The synergy of the three helps to improve the local immune microenvironment and reduce the recurrence risk. At the same time, ultrasound‐guided precise lesion resection ensures negative surgical margins and avoids recurrence caused by residual lesions, which is also an important reason why the recurrence rate of the OG was significantly lower than that of the CG.

The integrated strategy of “multitarget drug pretreatment–image‐guided precise clearance–immediate functional aesthetic reconstruction” advocated in this study represents an important direction in the evolution of NPM treatment from extensive management to refined and humanized treatment. Preliminary clinical evidence shows that this strategy can effectively synergize the advantages of different treatment methods. On the basis of ensuring surgical safety, it significantly reduces the risk of disease recurrence, greatly improves patients’ postoperative quality of life and mental health, and realizes the transformation of treatment goals from simply pursuing lesion clearance to a comprehensive treatment goal of “efficacy–aesthetics–psychology” trinity.

However, this study has several limitations. First, it was a single‐center retrospective analysis with nonrandomized group assignment; the use of a random number table was only for retrospective allocation to reduce bias, not prospective randomization. Second, the follow‐up period (median 16.8 months) is relatively short for a chronic, recurrent disease such as NPM; longer follow‐up (3–5 years) is needed to confirm the durability of the low recurrence rate observed. Third, the sample size is modest, and all patients were from a single institution in China, which may limit generalizability to other populations. Future directions should include the following: (1) multicenter, prospective randomized controlled trials with long‐term (5–10 years) follow‐up to validate durability of outcomes and impact on quality of life/lactation [38]; (2) development of refined classification systems (molecular/clinical‐imaging) to guide truly personalized therapy [39]; (3) exploration of minimally invasive techniques (e.g., microwave ablation) as potential neoadjuvant or alternative treatments [40, 41]; (4) deeper mechanistic studies to elucidate pathogenesis and drug synergies; and (5) formal implementation of multidisciplinary team (MDT) models to standardize and disseminate this integrated care approach.

## 5. Conclusion

In summary, the treatment of NPM should follow the principles of individualization, multimodality, and balancing efficacy with humanistic care, and conservative medical treatment is the first choice for mild NPM in the early solid or cavitary phase. The comprehensive pharmacotherapy (Xiaojin capsules + hormones + antibiotics) combined with ultrasound‐guided precise lesion resection + Stage I oncoplastic breast reconstruction plan adopted in this study is suitable for refractory NPM patients who fail conservative medical treatment or have extensive lesions/complications. This achieves the organic unity of disease treatment, cosmetic effect, and psychological rehabilitation through preoperative drug synergy to control inflammation and reduce lesions, intraoperative precise resection of lesions to preserve normal tissue, and postoperative Stage I oncoplastic reconstruction of breast appearance. Clinical data show that this plan not only significantly reduces the medium and long‐term recurrence rate of NPM patients (0% in the OG vs. 11.1% in the CG) but also significantly improves the breast appearance cosmetic effect and patient satisfaction, reduces the postoperative depression score, and does not increase the risk of complications or the length of hospital stay. It is a safe, effective, and integrated TCM–Western medicine treatment plan with both therapeutic and cosmetic values. This treatment strategy fully reflects the development trend of modern breast surgery, provides a new optimized option for the clinical treatment of NPM, and has important clinical promotion and application prospects. In the future, multicenter, large‐sample prospective studies with extended follow‐up (≥ 5 years) are needed to further verify its long‐term efficacy and safety.

## Author Contributions

Bao‐Zhong Yao conceived the study, designed and carried out the clinical work, and wrote the article. **Kun Peng**, **Sai-Long Sang**, and **Li Lin** participated in the data collection and interpretation (equal). **Hong-Cun Chen** and **Hong-Lin Li** took charge of postoperative pathologic examination and screening (equal). **Dai-Wei Shi** was involved in the review of literature. **Qi-Ru Xiong** and **Liang Li** took part in the revision of the article.

## Funding

This study was supported by the Traditional Chinese Medicine Research Project of Anhui Province (2024ZYYXH136) and Applied Medical Research Program of the Hefei Municipal Health Commission (Hwk2023zc001).

## Disclosure

All authors have read and approved the final manuscript.

## Ethics Statement

Ethical approval was granted by the Ethics Committee of Anhui Medical University Affiliated Hefei Hospital (2024‐科研‐147).

## Conflicts of Interest

The authors declare no conflicts of interest.

## Data Availability

All raw data and code are available upon request.

## References

[1] Kamal R. M. , Hamed S. T. , and Salem D. S. , Classification of Inflammatory Breast Disorders and Step by Step Diagnosis, Breast Journal. (August 2009) 15, no. 4, 367–380, 10.1111/j.1524-4741.2009.00745.x, 2-s2.0-67650769961. 19496780

[2] Zhou F. , Shang X. C. , Tian X. S. , Yu Z. G. , and Chinese Society of Breast Surgery , Clinical Practice Guidelines for Diagnosis and Treatment of Patients with Non-Puerperal Mastitis: Chinese Society of Breast Surgery (CSBrS) Practice Guideline 2021, Chinese Medical Journal. (May 2021) 134, no. 15, 1765–1767, 10.1097/CM9.0000000000001417. 34039865 PMC8367070

[3] Tan H. , Li R. , Peng W. , Liu H. , Gu Y. , and Shen X. , Radiological and Clinical Features of Adult Non-puerperal Mastitis, British Journal of Radiology. (April 2013) 86, no. 1024, 10.1259/bjr.20120657, 2-s2.0-84876034491. PMC3635790 23392197

[4] Co M. , Cheng V. C. C. , Wei J et al., Idiopathic Granulomatous Mastitis: A 10-Year Study From A Multicentre Clinical Database, Pathology. (December 2018) 50, no. 7, 742–747, 10.1016/j.pathol.2018.08.010, 2-s2.0-85055689002. 30389215

[5] Altintoprak F. , Kivilcim T. , Yalkin O. , Uzunoglu Y. , Kahyaoglu Z. , and Dilek O. N. , Topical Steroids are Effective in the Treatment of Idiopathic Granulomatous Mastitis, World Journal of Surgery. (November 2015) 39, no. 11, 2718–2723, 10.1007/s00268-015-3147-9, 2-s2.0-84943199556. 26148520

[6] Mizrakli T. , Velidedeoglu M. , Yemisen M. et al., Corticosteroid Treatment In The Management Of Idiopathic Granulomatous Mastitis To Avoid Unnecessary Surgery, Surgery Today. (April 2015) 45, no. 4, 457–465, 10.1007/s00595-014-0966-5, 2-s2.0-84938696058. 24993812

[7] Xue J. X. , Ye B. , Liu S. , Cao S. H. , Bian W. H. , and Yao C. , Treatment Efficacy of Chuang Ling Ye, a Traditional Chinese Herbal Medicine Compound, on Idiopathic Granulomatous Mastitis: A Randomized Controlled Trial, Evidence-Based Complementary and Alternative Medicine. (June 2020) 2020, no. 1, 10.1155/2020/6964801. PMC7341429 32714413

[8] Xu H. , Liu R. , Lv Y. et al., Treatments for Periductal Mastitis: Systematic Review and Meta-Analysis, Breast Care. (February 2022) 17, no. 1, 55–62, 10.1159/000514419. 35355704 PMC8914206

[9] Godazandeh G. , Shojaee L. , Alizadeh-Navaei R. , and Hessami A. , Corticosteroids in Idiopathic Granulomatous Mastitis: A Systematic Review and Meta-Analysis, Surgery Today. (December 2021) 51, no. 12, 1897–1905, 10.1007/s00595-021-02234-4. 33590327

[10] Sawuer R. , Wu C. , Sun Z. , and Liu S. , The Effectiveness of Traditional Chinese Medicine Combined with Surgery to Treat Granulomatous Mastitis: A Propensity-Matched Analysis, Frontiers Oncology. (February 2022) 12, 10.3389/fonc.2022.833742. PMC8866696 35223513

[11] Zhang C. , Wu Y. , Wang H et al., A Clinical Observation of Stage I Implant Breast Reconstruction for Mass-Like Granulomatous Lobular Mastitis, Gland Surgery. (September 2021) 10, no. 9, 2663–2672, 10.21037/gs-21-417. 34733716 PMC8514302

[12] Iwuchukwu O. C. , Harvey J. R. , Dordea M. , Critchley A. C. , and Drew P. J. , The Role of Oncoplastic Therapeutic Mammoplasty in Breast Cancer surgery-A Review, Surgical Oncology. (June 2012) 21, no. 2, 133–141, 10.1016/j.suronc.2011.01.002, 2-s2.0-84860350489. 21411311

[13] Urban C. , Lima R. , Schunemann E. , Spautz C. , Rabinovich I. , and Anselmi K. , Oncoplastic Principles in Breast Conserving Surgery, Breast. (October 2011) 20, no. 3, S92–S95, 10.1016/S0960-9776(11)70302-2, 2-s2.0-80054847669. 22015301

[14] Singh G. and Sharma R. K. , Immediate Breast Reconstruction for Phyllodes Tumors, Breast. (June 2008) 17, no. 3, 296–301, 10.1016/j.breast.2007.11.005, 2-s2.0-44349156065. 18155550

[15] Agha R. , Abdall-Razak A. , Crossley E et al., STROCSS 2019 Guideline: Strengthening the Reporting of Cohort Studies in Surgery, International Journal of Surgery. (2019) 72, 156–165, 10.1016/j.ijsu.2019.11.002. 31704426

[16] Agha R. A. , Mathew G. , Rashid R , Titan Group et al., Transparency in the Reporting of Artificial INtelligence-The Titan Guideline, Premier Journal of Science. (2025) 10, 10.70389/pjs.100082.

[17] Vandenbroucke J. P. , von Elm E. , Altman D. G et al., Strengthening the Reporting of Observational Studies in Epidemiology (STROBE): Explanation and Elaboration, Epidemiology. (2007) 18, no. 6, 805–835, 10.1097/ede.0b013e3181577511, 2-s2.0-35649024173. 18049195

[18] Zhou R. , Wu G. S. , He Y. K et al., Lesion Removal Plus Whole Breast Exploration And Washing Plus Micro-Plastic Procedures In The Treatment Of Granulomatous Lobular Mastitis: A Randomized Controlled Study, Zhonghua Wai Ke Za Zhi. (November 2021) 59, no. 11, 923–928, 10.3760/cma.j.cn112139-20201207-00845. 34743455

[19] Harris J. R. , Levene M. B. , Svensson G. , and Hellman S. , Analysis of Cosmetic Results Following Primary Radiation Therapy for Stages I and II Carcinoma of the Breast, International Journal of Radiation Oncology, Biology, Physics. (February 1979) 5, no. 2, 257–261, 10.1016/0360-3016(79)90729-6, 2-s2.0-0018383628. 110740

[20] Yin Y. , Le H. , Cheng Y. et al., A Cohort Study of Prolactin and Non-Puerperal Mastitis Using Real World Data, Scientific Reports. (March 2025) 15, no. 1, 10.1038/s41598-025-92504-9. PMC11903873 40075120

[21] Sripathi S. , Ayachit A. , Bala A. , Kadavigere R. , and Kumar S. , Idiopathic Granulomatous Mastitis: A Diagnostic Dilemma for the Breast Radiologist, Insights into Imaging. (August 2016) 7, no. 4, 523–529, 10.1007/s13244-016-0497-2, 2-s2.0-84983030852. 27164916 PMC4956625

[22] Zhang L. , Hu J. , Guys N et al., Diffusion-Weighted Imaging in Relation to Morphology on Dynamic Contrast Enhancement MRI: The Diagnostic Value of Characterizing Non-Puerperal Mastitis, European Radiology. (March 2018) 28, no. 3, 992–999, 10.1007/s00330-017-5051-1, 2-s2.0-85030112375. 28956122 PMC5811586

[23] Corona-López K. V. , Sánchez-Romero M. , Fernández-Rodríguez V. B. , Barba-Rojas A. K. , and Rendón-Molina A. , Variability in Clinical Response to Steroids in Granulomatous Mastitis: A Report of Two Cases, Cureus. (October 2025) 17, no. 10, 10.7759/cureus.93940. PMC12588367 41200645

[24] Yin Y. , Liu X. , Meng Q. , Han X. , Zhang H. , and Lv Y. , Idiopathic Granulomatous Mastitis: Etiology, Clinical Manifestation, Diagnosis and Treatment, Journal of Investigative Surgery. (March 2022) 35, no. 3, 709–720, 10.1080/08941939.2021.1894516. 33691563

[25] Işık F. , Pala E. , Alper F. et al., Skin Involvement of Idiopathic Granulomatous Mastitis: Sonographic, Clinical, and Histopathological Features, Breast Journal. (2025) 2025, no. 1, 10.1155/tbj/7224219. PMC12488297 41041149

[26] Alper F. , Abbasguliyev H. , Özmen S. , Yalçin A. , Yılmaz Çankaya B. , and Akçay M. N. , Clinical, Histopathological, Imaging, and Treatment Perspectives of Inflammatory Granulomatous Mastitis: Review of the Literature, Eurasian J Med. (December 2022) 54, no. 1, 172–178, 10.5152/eurasianjmed.2022.22306. 36655464 PMC11163346

[27] Davis J. , Cocco D. , Matz S. et al., Re-evaluating if Observation Continues to be the Best Management of Idiopathic Granulomatous Mastitis, Surgery. (December 2019) 166, no. 6, 1176–1180, 10.1016/j.surg.2019.06.030, 2-s2.0-85070238691. 31400951

[28] Lei X. , Chen K. , Zhu L. , Song E. , Su F. , and Li S. , Treatments for Idiopathic Granulomatous Mastitis: Systematic Review and Meta-Analysis, Breastfeeding Medicine. (September 2017) 12, no. 7, 415–421, 10.1089/bfm.2017.0030, 2-s2.0-85042626520. 28731822

[29] Montazer M. , Dadashzadeh M. , and Moosavi Toomatari S. E. , Comparison of the Outcome of Low Dose and High-Dose Corticosteroid in the Treatment of Idiopathic Granulomatous Mastitis, Asian Pacific Journal of Cancer Prevention. (April 2020) 21, no. 4, 993–996, 10.31557/APJCP.2020.21.4.993. 32334460 PMC7445984

[30] Papila K. B. , Velidedeoğlu M. , and Mete B. , The Effect of Methotrexate Monotherapy on treatment-resistant Idiopathic Granulomatous Mastitis Patients, The Surgeon. (June 2022) 20, no. 3, e13–e19, 10.1016/j.surge.2021.03.001. 33836950

[31] Liu L. , Zhou F. , Zhang X. et al., Granulomatous Lobular Mastitis: Antituberculous Treatment and Outcome in 22 Patients, Breast Care. (October 2018) 13, no. 5, 359–363, 10.1159/000487935, 2-s2.0-85049894961. 30498422 PMC6257198

[32] Li H. , Zhang G. , Wang H. et al., Ultrasound-Guided Microwave Ablation for the Treatment of Idiopathic Granulomatous Mastitis: Comparison with Surgical Excision, BMC Women’s Health. (April 2024) 24, no. 1, 10.1186/s12905-024-03070-7. PMC11025156 38637788

[33] Li Y. , Yang C. , Ding Y. et al., Clinical Effect of Ultrasound-Guided Microwave Ablation in the Treatment of 60 Patients With Non-Puerperal Mastitis, BMC Women’s Health. (May 2025) 25, no. 1, 10.1186/s12905-025-03804-1. PMC12102884 40410797

[34] Li C. , Wei X. , Wang Y. et al., Rotational Gland Dissection for Refractory Granulomatous Mastitis: A Single-Center Retrospective Study, Asian Journal of Surgery. (January 2024) 47, no. 1, 328–332, 10.1016/j.asjsur.2023.08.164. 37684121

[35] Kapoor N. S. , Ryu H. , Smith L. , Zou J. , Mitchell K. , and Blair S. L. , Presentation and Management of Granulomatous Mastitis in the United States: Results of an American Society of Breast Surgeons Registry Study, Annals of Surgical Oncology. (October 2024) 31, no. 11, 7396–7404, 10.1245/s10434-024-15714-x. 38969857 PMC11452424

[36] Huang Y. and Wu H. , A Retrospective Analysis of Recurrence Risk Factors for Granulomatous Lobular Mastitis in 130 Patients: More Attention Should be Paied to Prolactin Level, Annals of Palliative Medicine. (March 2021) 10, no. 3, 2824–2831, 10.21037/apm-20-1972. 33549007

[37] Seyidli C. , Aydoğdu Y. F. , Büyükkasap Ç. et al., The Role of Tissue IgG4 Levels in Steroid Therapy in Patients With Idiopathic Granulomatous Mastitis, Clinical and Experimental Medicine. (July 2024) 24, no. 1, 10.1007/s10238-024-01444-7. PMC11284177 39069567

[38] Yuan Q. Q. , Xiao S. Y. , Farouk O. et al., Management of Granulomatous Lobular Mastitis: An International Multidisciplinary Consensus (2021 Edition), Military Medical Research. (April 2022) 9, no. 1, 10.1186/s40779-022-00380-5. PMC9040252 35473758

[39] Uysal E. , Soran A. , Sezgin E. , and Granulomatous Mastitis Study Group , Factors Related to Recurrence of Idiopathic Granulomatous Mastitis: What do We Learn From a Multicentre Study?, ANZ Journal of Surgery. (June 2018) 88, no. 6, 635–639, 10.1111/ans.14115, 2-s2.0-85026301024. 28749045

[40] Zhou S. , Sheng C. , Hu P. et al., A Preliminary Study of Ultrasound-Guided Microwave Ablation for Nonpuerperal Mastitis Treatment, Breast Care. (February 2023) 18, no. 1, 1–11, 10.1159/000527128. 36876169 PMC9982351

[41] Wang Y. , Song J. , Tu Y. , Chen C. , and Sun S. , Minimally Invasive Comprehensive Treatment for Granulomatous Lobular Mastitis, BMC Surgery. (February 2020) 20, no. 1, 10.1186/s12893-020-00696-w. PMC7035639 32087717

